# Genome-Wide Analysis of the Thiamine Biosynthesis Gene Families in Common Bean Reveals Their Crucial Roles Conferring Resistance to Fusarium Wilt

**DOI:** 10.3390/biology14101366

**Published:** 2025-10-06

**Authors:** Ming Feng, Yu Liu, Yang Zhao, Tao Li, Jian Chen, Yuning Huang, Weide Ge, Chao Zhong, Renfeng Xue

**Affiliations:** 1 Crop Research Institute, Liaoning Academy of Agricultural Sciences, Shenyang 110161, China; 2 College of Agronomy, Shenyang Agricultural University, Shenyang 110886, China

**Keywords:** thiamine, Fusarium wilt, common bean, stress

## Abstract

Fusarium wilt, caused by *Fusarium oxysporum* f. sp. *phaseoli* (Fop), is a major constraint to global common bean (*Phaseolus vulgaris* L.) production. Thiamine (vitamin B1), an essential coenzyme in plant metabolism, has recently emerged as a potential regulatory factor in plant defense. Here, we performed a comprehensive genome-wide analysis of thiamine biosynthesis-related genes in common bean and elucidated their roles in resistance to Fusarium wilt. Five key thiamine biosynthetic genes were identified and characterized, showing conserved functional domains and evolutionary conservation across species. Expression profiling revealed tissue-specific patterns, with *PvTHI1-1* and *PvTHIC* being highly expressed in reproductive and photosynthetic organs, with their relative expression levels 0.28–0.57 higher than other members in the same tissue, while *PvTPK* maintained a basal expression level in the roots. Upon Fop infection, resistant genotypes exhibited significantly higher expression of thiamine biosynthetic genes and greater accumulation of endogenous thiamine and its derivatives than susceptible ones. Functional analysis using *Agrobacterium rhizogenes*-mediated transformation demonstrated that overexpression of *PvTPK* enhanced thiamine metabolism and conferred resistance in susceptible genotypes. Similarly, exogenous application of thiamine upregulated biosynthetic genes and improved disease resistance. Together, these results reveal that thiamine biosynthesis is intricately linked to Fusarium wilt resistance and that both genetic and biochemical manipulation of thiamine pathways can enhance disease tolerance. This study provides new insights into thiamine-mediated plant immunity and establishes a foundation for its application in the control of Fusarium wilt in common bean.

## Simple Summary

Fusarium wilt is a devastating soil-borne disease that can reduce common bean yields by up to 50% in severely affected regions worldwide. In this study, five key thiamine biosynthetic genes were identified in common bean, all showing evolutionary conservation across plant species. These genes exhibited distinct activity in different plant organs. Upon infection, resistant genotypes displayed 2–3 fold higher expression of thiamine biosynthetic genes and accumulated significantly greater amounts of thiamine and its active form ThDP compared with susceptible genotypes. Enhancement of the critical gene PvTPK in susceptible plants by biotechnology increased thiamine levels by about 1.8-fold and conferred improved resistance. Likewise, exogenous application of 50 mM thiamine induced defense-related genes and reduced wilt severity within 7 days. These findings demonstrate that natural thiamine metabolism plays a pivotal role in defense against Fusarium wilt and that both genetic enhancement and exogenous thiamine supplementation provide sustainable strategies to protect bean harvests from this disease.

## 1. Introduction

Common bean (*Phaseolus vulgaris* L.), belonging to the genus *Phaseolus* within the Fabaceae family, is a typical annual herbaceous plant. Due to its extensive ecological adaptability and exceptional nutritional value, it has emerged as the most widely cultivated edible legume crop globally [1,2,3]. Nevertheless, continuous threats posed by pests and diseases have severely hindered simultaneous advancements in yield and quality, with Fusarium wilt causing particularly significant damage [4,5]. Fusarium wilt of common bean is caused by the soil-borne pathogenic fungus *Fusarium oxysporum* f. sp. *phaseoli* (Fop), which colonizes the vascular system of the root, leading to wilting, leaf chlorosis, and ultimately plant death [6]. The pathogen was first reported in California in 1929 and subsequently identified in major bean-producing regions worldwide, including the Americas, Africa, Asia, and Europe [7,8]. Fusarium wilt is considered one of the most destructive diseases of legumes, with yield losses frequently ranging from 30 to 50% in severely infested fields [9,10]. In tropical Africa and Latin America, the disease is widespread and significantly reduces farmer income and food security [11]. Recent reports also emphasize its increasing prevalence under intensive cropping systems and changing climate conditions [8]. Agricultura fields characterized by high moisture conditions, dense planting patterns, and inadequate crop rotation practices are particularly prone to this disease [10]. Currently, the most efficient and environmentally sustainable strategy for managing Fusarium wilt involves identifying resistance genes in common bean, elucidating their mechanisms of resistance, and integrating these genes into breeding programs for improved resistance. Consequently, research focused on its effective prevention and control is of substantial economic importance and scientific significance.

Thiamine (vitamin B1, VB1), as an essential water-soluble vitamin for organisms, has dual core functions in maintaining life activities: its chemical essence is colorless crystalline vitamin B1, which is not only a key coenzyme factor for cellular energy metabolism [12], but also an important signal molecule for regulating growth and development [13]. The biosynthetic pathway of this compound shows significant species specificity-fungi, bacteria and plants synthesize it autonomously through a conserved thiamine synthesis pathway, while animals and humans rely on exogenous intake to meet physiological needs [14]. It is noteworthy that this evolutionary difference in synthetic ability allows thiamine to serve as both a nutritional factor and a potential defense regulatory role in the plant-animal interaction system. The metabolic homeostasis of thiamine in plants presents polymorphic characteristics, which mainly include three active forms: free thiamine, thiamine monophosphate (TMP) and thiamine pyrophosphate (TPP) [14]. It is worth noting that TPP, as the main functional form, not only affects energy metabolism by regulating photosynthetic phosphorylation and the tricarboxylic acid cycle [15], but its dynamic balance also directly determines chloroplast development. When thiamine levels are insufficient, it will lead to blocked protein synthesis in the photoreaction center, imbalance in amino acid metabolism and accumulation of carotenoid precursors, ultimately causing characteristic leaf chlorosis symptoms [16].

The biosynthetic pathway of thiamine follows a multi-enzyme synergistic catalytic mechanism, the core of which is dominated by a cascade reaction network composed of four key enzymes. Firstly, phosphomethylpyrimidine synthase (THIC) uses 5-aminoimidazole ribonucleotide (AIR) as a substrate and, under the regulation of cofactors S-adenosylmethionine (SAM) and NADH, catalyzes the production of 4-amino-2-methyl-5-hydroxymethylpyrimidine monophosphate (HMP-P), completing the de novo synthesis of the pyrimidine ring structural unit [17]. Secondly, thiazole synthase (THI1) converts nicotinamide adenine dinucleotide (NAD) and glycine (Gly) into adenylated thiazole intermediate (ADT) through a sulfur atom transfer reaction, achieving precise assembly of the thiazole ring module [18]. Subsequently, thiamine phosphate synthase (TH1) acts as a molecular coupling hub to condense the pyrimidine precursor HMP-PP with the thiazole precursor HET-P in an ATP-dependent manner to generate thiamine monophosphate (ThMP) [19]. Finally, thiamine pyrophosphokinase (TPK) catalyzes the γ-phosphate transfer of thiamine diphosphate (ThDP) through the Mg^2+^-ATP energy supply system in the cytosol to form the metabolically active coenzyme form TPP and regulates its targeted transport to chloroplasts and mitochondria [20]. Studies have shown that the loss-of-function mutants of the above four enzymes in *Arabidopsis thaliana* all exhibited embryonic lethality or severe growth arrest phenotypes, and the reversible replenishment experiment of exogenous thiamine, confirmed the irreplaceable nature of this enzyme system in the plant thiamine metabolic network and its potential value as a key target in the metabolic process [19].

Recent studies have shown that thiamine, as an endogenous defense regulatory factor in plants, participates in both biotic and abiotic stress responses through a dual stress resistance mechanism [21,22,23,24,25]. In terms of biotic stress, it significantly enhances host disease resistance by activating the systemic acquired resistance (SAR) signaling pathway, inducing pathogenesis-related protein (PR protein) gene expression and H_2_O_2_-mediated reactive oxygen burst [26]. Related studies have confirmed that exogenous thiamine treatment can increase the resistance of crops such as rice, *Arabidopsis* and tobacco to infection by fungi (such as rice blast fungus), bacteria (such as Pseudomonas syringae) and viruses (TMV) by 30–50% [26,27]. As an important regulatory factor in plant stress resistance, the thiamine gene family has been confirmed to respond to biotic and abiotic stresses in a variety of crops [28,29,30]. Although exogenous thiamine has been proven to stimulate systemic resistance of model plants such as rice and Arabidopsis [26,27], and the function of its synthesis of key genes (such as *AtTHIC*, *AtTPK*) has been studied in model species [19,20], there is still a huge gap in its functional mechanism in the response to Fusarium wilt disease. At present, there is a lack of systematic identification of the thiamine metabolic gene family, and the expression patterns and functions of the family members under the stress of soil-transmitted vascular disease are unknown. In this study, we used bioinformatics methods to comprehensively identify and analyze the thiamine gene family in the common bean genome. Furthermore, we explored the expression pattern of this gene family under the stress of Fusarium wilt. In addition, functional verification of the key genes was performed using *Agrobacterium rhizogenes*-mediated root transformation. This study revealed for the first time the phylogenetic law of the thiamine gene family in common beans, which not only provided a theoretical basis for analyzing the disease resistance mechanism of Fusarium wilt, but also laid the foundation for related disease resistance molecular breeding.

## 2. Materials and Methods

### 2.1. Plant Materials and Fop Inoculation

Common bean genotypes BRB130 (Fusarium wilt susceptible) and CAAS260205 (Fusarium wilt resistant) were obtained from the Institute of Crop Sciences of the Chinese Academy of Agricultural Sciences (CAAS) in Beijing, China [31,32,33]. An aggressive *F. oxysporum* f. sp. *phaseoli* (Fop) isolate, FOP-DM01 (GenBank accession number: HM756257.1) was used for the experiments as further described by Xue et al. [34]. Seedlings of BRB130 and CAAS260205, grown in a greenhouse for 10 days following the method described by Xue et al. [31], were inoculated at the seedling stage when the first true leaves were fully expanded. Resistant and susceptible genotypes were grown separately, each in 10 pots containing a single plant. For pathogen inoculation, the FOP-DM01 isolate was first propagated in a cornmeal–vermiculite (CVM) substrate. The colonized CVM was then blended thoroughly with a sterilized soil medium composed of clay and vermiculite (1:3, *v*/*v*), resulting in an inoculum concentration of approximately 5.0 × 10^6^ colony-forming units (cfu) per gram of soil. Seedlings were subsequently transplanted into pots filled with the inoculated soil mixture to ensure uniform infection conditions. Leaf, stem, and root tissues were collected and stored at −80 °C, and samples taken at 0, 48, and 96 h after inoculation were used to analyze the expression of related genes and the content of endogenous thiamine.

To explore responses to thiamine, leaves of the Fusarium wilt-susceptible genotype BRB130 were sprayed with 50 mM thiamine diluted in ddH_2_O. Plants were cultivated under controlled greenhouse conditions, where temperatures fluctuated between approximately 22 °C and 28 °C. All experimental treatments were maintained for 14 days under consistent conditions, including exposure to natural sunlight supplemented with artificial lighting. For the *Fusarium oxysporum* f. sp. *phaseoli* (Fop) treatment, samples of leaf, stem, and root tissues were collected at 0, 48, and 96 h post-inoculation (hpi). All harvested tissue samples were immediately frozen and preserved at −80 °C until further analysis.

### 2.2. Identification of Key Gene Family Members in the Thiamine Biosynthesis Pathway of Phaseolus vulgaris

The genome data of common bean were downloaded from the Phytozome Databases (https://phytozome-next.jgi.doe.gov/, accessed on 16 January 2025). First, the consensus conservative seed files of Hidden Markov models (HMMs) of the key gene families *THIC*, *TH1*, *THI1*, and *TPK* in the thiamine biosynthesis pathway were downloaded from the Pfam website1 (PF01964, PFPF08543, PF02581, PF01946, PF04263, and PF04265). Then, the HMM profile was performed as a query to identify all specific Thiamine-containing domain in common bean by retrieving against the genome with a threshold of e-value of <e^−3^. The same method was used to screen out key genes in thiamine biosynthesis pathway in *Glycine Max*, *Cicer arietinum*, *Oryza Sativa*, *Zea mays*, *Sorghum bicolor*, *Vitis vinifera*, *Medicago truncatula*. Information on key genes in thiamine biosynthesis pathway family membership in *Arabidopsis* was obtained from published articles [17,18,20,35]. Then, the sequences of potential proteins were submitted to SMART (https://smart.embl.de/, accessed on 25 January 2025) and NCBI CDD (https://www.ncbi.nlm.nih.gov/cdd/, accessed on 25 January 2025) to search for the specific Thiamine domains and removed the non-representative transcripts. The protein sequences of the identified members of the thiamine biosynthesis gene family were submitted to Expasy website (https://web.expasy.org/, accessed on 26 January 2025) to calculate theoretical PI and molecular weight of the proteins coded by these *PvTHIC*, *PvTH1*, *PvTHI1* and *PvTPK* genes. The subcellular location of *PvTHIC*, *PvTH1*, *PvTHI1* and *PvTPK* was PSORT website (https://wolfpsort.hgc.jp, accessed on 26 January 2025).

### 2.3. Chromosome Location Analysis and Gene Structure Analysis

The chromosome length and location information of the five thiamine biosynthesis gene family members were extracted from the common bean genome file and annotation file. Then the TBtools program was used to visualize the target genes on the chromosomes [36].

### 2.4. Phylogenetic Analysis and Collinearity Analysis of Key Genes in the Common Bean Thiamine Biosynthesis Pathway

To study the divergence and evolutionary relationship of the common bean thiamine biosynthesis family genes with those from other plant species, we constructed a phylogenetic tree among the protein sequences of the thiamine family members of common bean and those of other species, including *Arabidopsis thaliana*, *Glycine Max*, *Cicer arietinum*, *Arachis hypogaea*, *Oryza sativa*, *Zea mays*, *Sorghum bicolor*, *Vitis vinifera*, and *Medicago truncatula*. The thiamine protein sequences of nine species including common bean were aligned by ClusterW method (default parameter) using the software MEGA11 software [37]. The Maximum Likelihood (ML) method was used to construct a phylogenetic tree, test of phylogeny as bootstrap method 1000, model/method as Poisson model. After preliminary construction of the evolutionary tree, the EvolView website (www.evolgenius.infi/evolview/#/terrview, accessed on 8 February 2025) was used for further beautification [38,39]. FASTA genomic data files and GFF3 gene annotation files of *Arabidopsis thaliana*, *Phaseolus vulgaris*, *Glycine Max*, *Cicer arietinum*, *Oryza Sativa*, *Zea mays*, *Sorghum bicolor*, *Vitis vinifera*, and *Medicago truncatula* were downloaded from the Phytozome Database (https://phytozome-next.jgi.doe.gov/, accessed on 16 January 2025). Based on the genome sequence and gene annotation files, the collinearity files between every two species, including collinearity and collinear gene pairs, were obtained using the one-step MCScanX plug-in in TBtools. The Ka and Ks values of the obtained orthologous gene pairs were used to calculate the ka/ks ratio of all the homologous gene pairs [40].

### 2.5. Conserved Cis-Elements Analysis in Promoters

The common bean genome database was employed to extract a 2000 bp sequence upstream of the initiation codon of common bean thiamine genes to act as the promoter region. The *cis*-acting element existence in the gene was assessed via the PlantCARE database (http://bioinformatics.psb.ugent.be/webtools/plantcare/html/, accessed on 5 July 2025), which is dedicated to the study of these elements in plants. A regulation prediction tool, the Plant Transcriptional Regulatory Map (PTRM) (http://plantregmap.gao-lab.org/, accessed on 13 July 2025) was used to infer potential regulatory interactions of TFs in the upstream (2000 bp) regions of common bean thiamine genes with threshold (*p*-value ≤ 1 × 10^−7^). *Arabidopsis* is used as a reference species.

### 2.6. Tissue-Specific Expression Analysis of Thiamine Biosynthesis Genes Based on RNA-Seq Data

Publicly available transcriptome data for *Phaseolus vulgaris* were used to analyze the tissue-specific expression patterns of key thiamine biosynthesis genes. RNA-seq datasets encompassing 11 distinct tissues across different developmental stages—namely flower buds, flowers, green mature pods, leaves, young trifoliates, nodules, roots at 10 and 19 days, and stems at 10 and 19 days—were retrieved from the Phytozome v13 genome database. Gene expression levels were quantified as fragments per kilobase of transcript per million mapped reads (FPKM), and subsequently transformed to log_2_ (FPKM) values for visualization. Heatmap generation and hierarchical clustering were performed using the TBtools software (TBtools-II v2.326) platform to assess spatial expression variation in thiamine biosynthetic genes across the sampled tissues.

### 2.7. Analysis of Thiamine and Its Phosphate Analogs

Two common bean genotypes, BRB130 and CAAS260205, were utilized to investigate changes in endogenous thiamine and its phosphorylated derivatives following inoculation with *Fusarium oxysporum* f. sp. *phaseoli* (Fop). Root, stem, and leaf tissues were separately harvested at 96 h post-inoculation (hpi), flash-frozen in liquid nitrogen, and stored at −80 °C. Thiamine, thiamin monophosphate (ThMP), and thiamine diphosphate (ThDP) contents were quantified using high-performance liquid chromatography (HPLC). Each treatment included three independent biological replicates. Data obtained were statistically analyzed to assess genotype and tissue-specific differences in thiamine metabolism following pathogen infection.

### 2.8. qPCR Analysis

Total RNA was isolated from all examined tissues using the Plant Total RNA Extraction Kit (Tiangen Biotech, Beijing, China), following the manufacturer’s protocol. First-strand cDNA synthesis was performed using the PrimeScript™ RT Reagent Kit (TaKaRa, Japan) under the recommended conditions. Gene-specific primers targeting key thiamine biosynthesis genes were designed via Primer-BLAST (https://www.ncbi.nlm.nih.gov/tools/primer-blast/, accessed on 1 August 2025). Quantitative real-time PCR was conducted using the SYBR Premix Ex Taq II (Tli RNaseH Plus) kit (TaKaRa, Japan), with Actin11 employed as the internal reference gene. Fluorescence detection and amplification were carried out on an ABI 7500 Real-Time PCR System (Applied Biosystems, USA). Relative gene expression levels were calculated using the 2^−ΔΔCT^ method [41], based on three biological replicates per sample.

### 2.9. Construction of the PvTPK Overexpression Vector and A. rhizogenes-Mediated Transformation

The overexpression and gene silencing vectors of *PvTPK* were constructed by introducing XbaI and SacI restriction sites at the upstream and downstream ends of the *PvTPK* coding sequence, respectively, using primers OE-F and OE-R (Table S1). The *PvTPK* cDNA was digested with XbaI and SacI, and subsequently ligated into the p35SGFPGUS+ vector that had been digested with the same enzymes. The resulting overexpression construct was designated as p35S-PvTPK. Induction of transgenic hair roots was performed following the method described by Estrada-Navarrete et al. [42]. The generation, selection, and inoculation of transgenic roots were conducted according to protocols established by [31,32]. Phenotypic observations were carried out at 96 h post-inoculation. Leaf, stem, and root tissues were collected at 0, 48, and 96 h after inoculation for the analysis of gene expression and endogenous thiamine content.

### 2.10. Data Analysis

Statistical analyses were carried out in R version 4.3.0. For comparisons among multiple treatments, analysis of variance (ANOVA) was performed using the aov function. When only two groups were compared, Student’s *t*-tests were conducted with the t.test function. Statistical significance was considered at *p* < 0.05, and highly significant differences at *p* < 0.01. Figures were generated using the ggplot2 4.0.0 package in R.

## 3. Results

### 3.1. Identification and Gene Structure Analysis of Key Gene Family Members of Phaseolus vulgaris Thiamine

**Figure 1:**
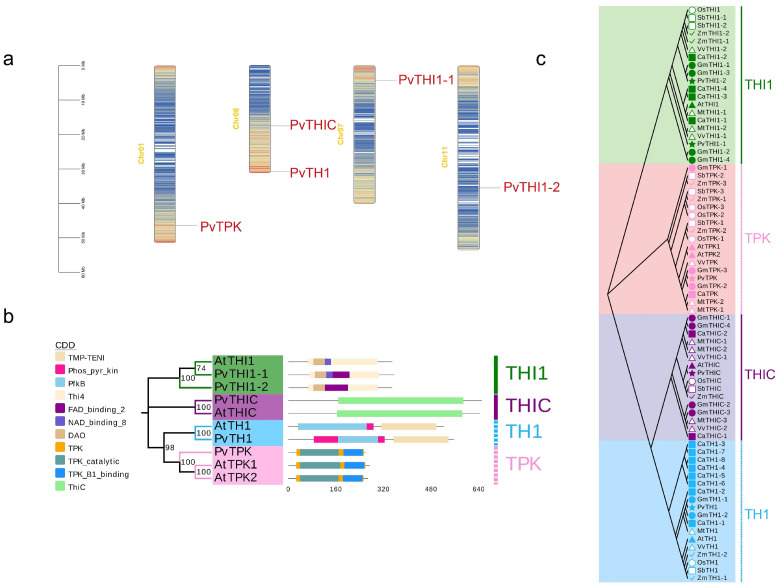
Chromosome, conserved domain and phylogenetic tree of the thiamine genes in common bean and *Arabidopsis*. (**a**) Distribution of thiamine genes in common bean chromosome. (**b**) The conservative domain of common beans. (**c**) Phylogenetic tree of the thiamine protein sequence in common bean and other species constructed by the maximum likelihood (ML) method.

HMMs of *THI1* (PF01946), *TPK* (PF04263 and PF04265), *THIC* (PF01964) and *TH1* (PF08543 and PF02581) were used to search the common bean genome. Finally, five members were identified in common bean (Table 1). In common bean, *THI1*, *TPK*, *THIC* and *TH1* genes have 2, 1, 1 and 1 members, respectively. The *THI1* gene is distributed on chromosomes 7 and 11, the *TPK* gene is distributed on chromosome 1, and the *THIC* gene and *TH1* gene are distributed on chromosome 6 (Figure 1a). All sequences were analyzed for domains using Pfam, SMART and CDD websites. The results showed that the common bean *THI1* gene sequences all contained Thi4, DAO and FAD binding 2 domains, while *PvTHI1-1* also contained NAD binding 8 domains. The *PvTPK* sequence contained TPK, TPK catalytic and TPK B1 binding domains. The *PvTHIC* sequence contained the Thic domain. The *PvTH1* sequence contains PfkB, Phos_pyr_kin, and TMP-TENI domains. Except for the differences in the conserved domains between *PvTHI1* and *Arabidopsis* members, the conserved domains of other common bean members and *Arabidopsis* members are consistent (Figure 1b). The number of amino acids, molecular weight (MW) of the encoded proteins, subcellular localization, and isoelectric point (pI) of the candidate *PvTHI1*, *PvTPK*, *PvTHIC*, and *PvTH1* were further analyzed (Table 1). The coding lengths of the five common bean members ranged from 650 (*PvTHIC*) to 259 (*PvTPK*) amino acids, and the number of amino acids in the two PvTHI1 members was similar, 348 and 355 amino acids, respectively. The molecular weights (kDa) of the predicted PvTHI1 proteins were 37.83 and 36.75, and the isoelectric points (pI) were 5.93 and 5.85, respectively. The amino acid numbers of *PvTPK*, *PvTHIC* and *PvTH1* are 28.91, 72.41 and 59.64, respectively, and the molecular weights are 6.24, 6.1 and 7.55, respectively. Subcellular localization prediction of the five protein members showed that except for *PvTPK*, which was located in the nucleus and cytoplasm, the other four members were located in the chloroplasts.

### 3.2. Phylogenetic Evolution and Collinearity Analysis of Key Gene Families of Phaseolus vulgaris Thiamine

In order to clarify the evolutionary relationship among different species of *THI1*, *TPK*, *THIC* and *TH1* genes in kidney bean, the phylogenetic tree of common bean and other legumes, grasses and dicots was constructed by maximum likelihood (ML) method. As shown in Figure 1c, there are 73 members in common bean, chickpea (*Cicer arietinum*), soybean (*Glycine max*), alfalfa (*Medicago sativa*), sorghum (*Sorghum bicolor*), rice (*Oryza sativa*), corn (*Zea mays*), *Arabidopsis* and grape (*Vitis vinifera*) (Table S2), which are divided into four subfamilies based on the classification results of *Arabidopsis* THI1, TPK, THIC and TH1 proteins. The THI1 subfamily contains 20 members, the TPK subfamily contains 19 members, the THIC subfamily contains 16 members, and the TH1 subfamily contains 18 members.

**Figure 2:**
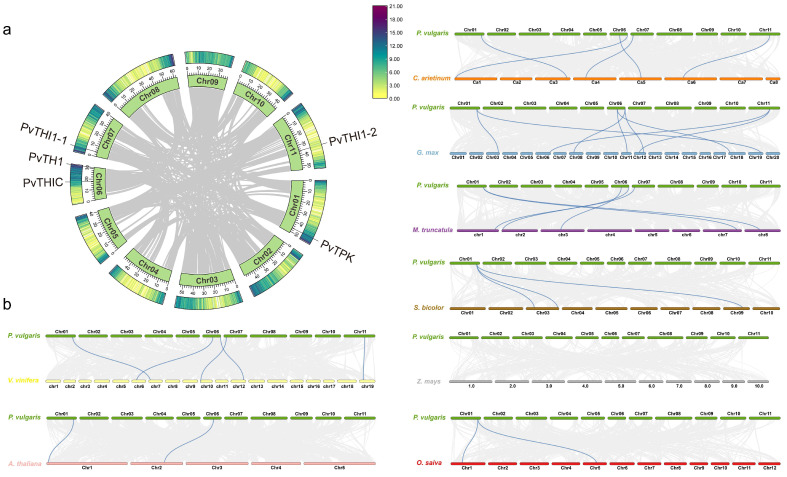
Syntenic analysis of thiamine-related genes in common bean and other plant species. (**a**) Intraspecies synteny of thiamine genes within the common bean genome. (**b**) Comparative synteny of thiamine genes between common bean and eight other species: soybean, chickpea, alfalfa, *Arabidopsis*, grape, rice, sorghum, and maize. Gray lines represent general collinear regions among genomes, while blue lines highlight syntenic thiamine gene pairs.

To further explore the evolutionary relationship between common bean and other species, the relationships of the four gene family members of common bean and the orthologous relationships with other species were studied. Orthologous gene pairs of *THI1*, *TPK*, *THIC* and *TH1* genes of common bean and other plants were also identified (Figure 2). Common beans and other legumes, including soybeans, alfalfa and chickpeas, have 8, 5 and 5 gene pairs, respectively; dicots grapes and *Arabidopsis* have 5 and 2 gene pairs with beans, respectively; and grasses rice, sorghum and corn have 2, 3 and 0 gene pairs with beans, respectively. Collinearity analysis showed that common bean and soybean are the closest relatives. By comparing the orthologous gene pairs, it was found that the orthologous relationships of the *THI1*, *TPK*, *THIC*, and *TH1* genes varied among different species. For example, within the THI1 subfamily, *PvTHI1-1* and *PvTHI1-2* have 3 and 4 homologous gene pairs with other species, respectively. In comparison, *PvTPK* in the TPK subfamily has 12 homologous gene pairs, *PvTHIC* in the THIC subfamily has 6, and *PvTH1* in the TH1 subfamily has 5 homologous gene pairs (Table S3). The *THI1*, *TPK*, *THIC* and *TH1* genes of common bean are homologous to soybean, chickpea, alfalfa and grape, while common bean and *Arabidopsis* are homologous only in the THIC and TPK subfamilies, and grass crops are homologous only in the TPK subfamily. To analyze the evolutionary selection pressure of thiamine biosynthesis pathway gene family genes, we calculated the Ka/Ks ratios of *THI1*, *TPK*, *THIC* and *TH1* gene pairs. Except for 2 colinear gene pairs that could not be calculated, the Ka/Ks ratios of the remaining 30 colinear gene pairs between common bean and other species were all less than 1, indicating that the *THI1*, *TPK*, *THIC* and *TH1* gene families were mainly subject to purifying selection during evolution (Table S4).

### 3.3. Analysis of Cis-Acting Elements of Thiamine Biosynthesis Gene

**Figure 3:**
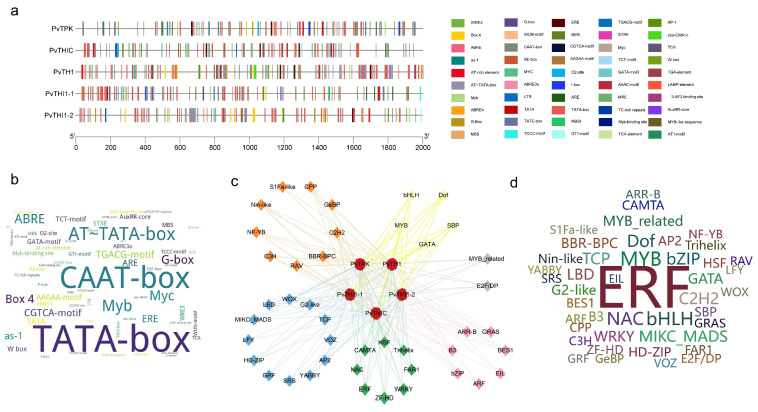
*Cis*-element and transcription factor (TF) analysis of thiamine biosynthesis gene promoters. (**a**) Distribution of cis-regulatory elements in the promoter regions of five thiamine biosynthesis genes. (**b**) Word cloud illustrating *cis*-elements, where larger font size indicates higher frequency. (**c**) Predicted TF regulatory network associated with thiamine biosynthesis. Diamonds represent TFs, while circles denote thiamine biosynthesis genes. Circle colors correspond to TF functional categories: blue (growth and development), green (stress response), pink (hormone signaling), yellow (metabolic regulation), orange (cell cycle and proliferation), gray (specialized functions), and orange (unspecified). (**d**) Word cloud of TFs, with font size proportional to the number of associated genes.

We predicted a total of 893 potential *cis*-acting elements in promoter regions, encompassing 50 functional categories (Figure 3a,b, Table S6). These elements further elucidate the potential contributions of thiamine to plant growth and development, hormone responses, and stress responses (Table S6). Specific analysis identified 20 growth- and development-related elements (e.g., TATA-box, CAAT-box, Box 4, O2-site), 12 hormone signaling-related elements (involving abscisic acid ABRE, jasmonic acid CGTCA-motif/TGACG-motif, ethylene ERE, and salicylic acid TCA/as-1 responses), and 18 stress-responsive elements (e.g., MYB, MYC, LTR, W box, TC-rich repeats, WUN-motif). The number and distribution of these elements varied across gene promoters, with *PvTHI1-1* having the highest number (28) and *PvTHIC* having the lowest number (21). Special attention was paid to biotic stress response elements (including W box, WUN-motif, TC-rich repeats, as-1, ERE, TGACG-motif, CGTCA-motif, WRE3, LTR), among which ERE was distributed in all genes. *PvTPK* (20 elements) and *PvTH1* (12 elements) contained the most biotic stress response elements. *PvTPK* has core defense elements (as-1(4), TGACG-motif(4), CGTCA-motif(4)) and unique WUN-motif and WRE3. *PvTH1* contains the key pathogen response element W box. These results indicate that the promoter of the bean thiamine biosynthesis gene has rich cis-acting element diversity, and its functional expression is complexly regulated by multiple elements closely related to hormone signaling, defense response and damage response.

Analysis of transcription factors in the promoter regions of thiamine biosynthesis genes revealed a total of 5094 transcription factors belonging to 44 families, including those involved in stress response, such as ERFs, NACs, and WRKYs; those involved in hormone signaling, such as ARFs and BES1; those involved in growth and development regulation, such as MIKC_MADS and LBDs; and other families involved in metabolic regulation (Figure 3c, Table S7). Among the predicted transcription factors, the ERF family was the most numerous (1904), followed by MYBs (333), bHLHs (278), NACs (263), and Dofs (217) (Table S6). Prediction indicated that the ERF family is the most important transcription factor family regulating thiamine biosynthesis in common bean, suggesting a key role in pathogen defense. Furthermore, the stress response-related NAC and WRKY families also exhibited multiple regulatory mechanisms on their members (Figure 3d). A regulatory network of transcription factors constructed based on the predictions further revealed functional associations between members in plant growth, stress response, and hormone signaling.

### 3.4. Expression Profiling of Thiamine Biosynthesis-Related Genes in Different Tissues

**Figure 4:**
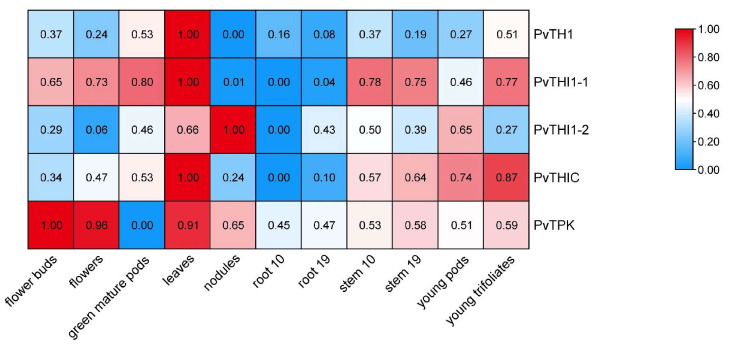
Expression profiles of thiamine biosynthesis-related genes in different tissues of *Phaseolus vulgaris*. A heatmap showing the transcript abundance (FPKM values) of five thiamine biosynthesis-related genes (*PvTHI1*, *PvTHI1-1*, *PvTHI1-2*, *PvTHIC*, and *PvTPK*) across 11 different tissues of common bean. Expression levels were log_2_-transformed and visualized by color gradient.

To explore the spatial expression patterns of thiamine biosynthesis-related genes in common bean, we analyzed transcriptome data across 11 different tissues, including flower buds, green mature pods, flowers, leaves, nodules, roots (10 and 19 days), stems, and young trifoliates. *PvTHI1-1* exhibited the highest expression in floral organs, early-stage pods, and leaves, indicating its possible involvement in reproductive development and photosynthetic tissues (Figure 4). *PvTHI1-2* showed a more tissue-specific pattern, with predominant expression in nodules, suggesting a potential role in nodule function or nitrogen fixation. *PvTHIC* was moderately expressed in multiple tissues, particularly enriched in stems and early pod development stages, implying its participation in both vegetative and reproductive growth. In contrast, both *PvTHI1* and *PvTPK* displayed generally low expression levels across all tissues, with no strong tissue-specific preference. Nonetheless, their expression was still detectable in most organs, including roots, indicating basal transcriptional activity under normal physiological conditions.

### 3.5. Differential Analysis of Thiamine Biosynthesis Gene Expression and Endogenous Thiamine Content After Fusarium Wilt Inoculation Between Resistance and Susceptible Genotypes

**Figure 5:**
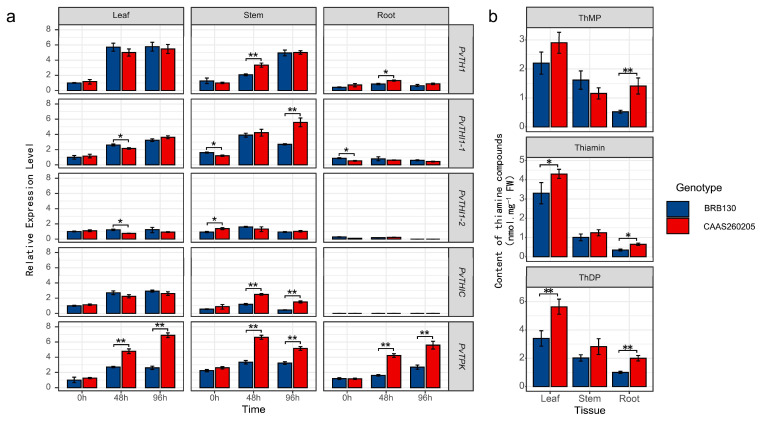
Differential expression of thiamine biosynthesis genes and endogenous thiamine compound content in resistant (CAAS260205) and susceptible (BRB130) genotypes following Fusarium wilt inoculation. (**a**) Relative expression levels of five thiamine biosynthesis-related genes (*PvTHI1*, *PvTHI1-1*, *PvTHI1-2*, *PvTHIC*, and *PvTPK*) in leaf, stem, and root tissues of BRB130 (blue) and CAAS260205 (red) at 0, 48, and 96 h post inoculation (hpi). (**b**) Quantification of three thiamine-related compounds—thiamine monophosphate (ThMP), thiamine (free form), and thiamine diphosphate (ThDP)—in leaf, stem, and root tissues of both genotypes. All gene expression levels were normalized and are shown as relative expression levels. Data represent mean ± SD (n = 3). Statistical differences between genotypes at the same time point or tissue were analyzed using a two-tailed Student’s *t*-test. Asterisks indicate significant differences (*, *p* < 0.05; **, *p* < 0.01).

To investigate the role of thiamine metabolism in Fusarium wilt resistance, we analyzed the expression of five key thiamine biosynthesis genes and quantified endogenous thiamine-related compounds in resistant (CAAS260205) and susceptible (BRB130) common bean genotypes following pathogen inoculation. The expression levels of *PvTHI1*, *PvTHI1-1*, *PvTHIC*, and *PvTPK* were significantly higher in CAAS260205 than in BRB130 in various tissues at 48 and 96 hpi (Figure 5a). Specifically, the expression amount of *PvTHI1* in CAAS260205 leaves was about 2.5 times that of BRB130, about 3.2 times at the stem, and about 2.8 times at the root; by 96 h, the leaves and roots remained high expression of about 2 times more than 2 times. Notably, *PvTHI1-1* showed a marked upregulation in stem and root tissues of the resistant genotype at 96 hpi (4.1 times and 3.5 times of BRB130, *p* < 0.01). Similarly, the stem expression level of *PvTHIC* at 96 h was about 3.8 times that of BRB130 and about 3.2 times at the root; *PvTPK* was upregulated about 3.3 times and 2.9 times in the stem and root, respectively, showing a strong inducible effect. Consistent with the gene expression data, quantitative analysis of thiamine metabolites (Figure 5b) showed that the content of thiophylic acid (ThMP), thiamine and thiophylic acid (ThDP) in the leaf tissue of CAAS260205 was significantly higher than that of BRB130 at 48 h and 96 h, with ThMP increased by about 35% and 42%, thiamine increased by about 50% and 55%, and ThDP increased by about 60% and 65%, respectively. Particularly, ThDP accumulation was significantly elevated in leaves and roots of the resistant genotype(roots increase by about 40% and 50%), while thiamine levels in roots and stems were also notably higher. These results suggest that enhanced thiamine biosynthesis and accumulation may contribute to the resistance mechanism against Fusarium wilt in CAAS260205.

### 3.6. Exogenous Thiamine Application and PvTPK Overexpression Enhance Resistance to Fusarium Wilt in the Susceptible Genotype

**Figure 6:**
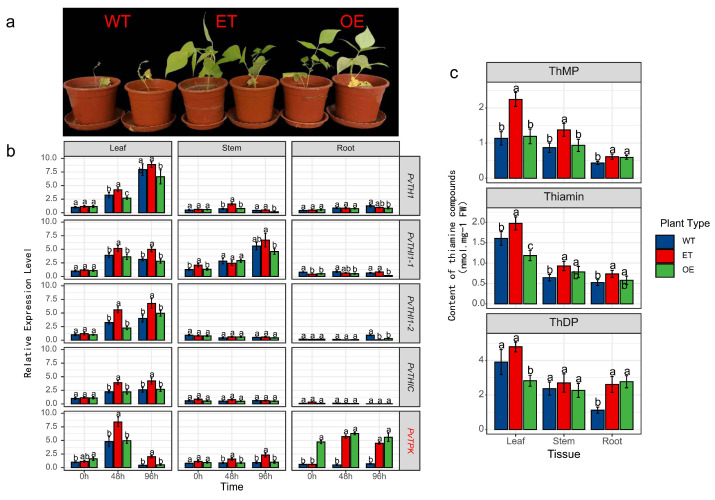
Effects of exogenous thiamine application and *PvTPK* overexpression on resistance to Fusarium wilt in the susceptible genotype BRB130. (**a**) Phenotypes of wild type (WT), exogenous thiamine-treated (ET), and *PvTPK* overexpression (OE) plants at 7 days post-inoculation with Fusarium oxysporum f. sp. phaseoli (Fop). (**b**) Relative expression levels of thiamine biosynthesis-related genes (*PvTHI1*, *PvTHI1-1*, *PvTHI1-2*, *PvTHIC*, and *PvTPK*) in leaf, stem, and root tissues of WT, ET, and OE plants at 0, 48, and 96 h post-inoculation. (**c**) Content of thiamine-related compounds-thiamine monophosphate (ThMP), thiamine, and thiamine diphosphate (ThDP)-in different tissues (leaf, stem, and root) of the three plant types. All data are presented as mean ± SD (n = 3). Statistical significance was determined using one-way ANOVA followed by Duncan’s multiple range test. Different letters indicate significant differences between treatments at *p* < 0.05.

To further verify the role of thiamine in resistance to Fusarium wilt in common bean, exogenous application of thiamine and *PvTPK* overexpression via *Agrobacterium rhizogenes*-mediated transformation were applied to the susceptible genotype BRB130, followed by phenotypic, transcriptional, and metabolic analyses under inoculated conditions. Exogenous application of thiamine (ET) and overexpression of the *PvTPK* gene (OE) both significantly enhanced resistance to Fusarium wilt in common bean, as evidenced by improved growth and reduced disease symptoms compared to wild-type (WT) plants (Figure 6a). Gene expression analysis showed that all tissues of OE plants showed significant upregulation of *PvTPK* at all time points, with an expression of approximately 3.5–4.8 times that of wild type (Figure 6b). Additionally, the expression of upstream biosynthesis genes such as *PvTHI1* and *PvTHIC* was significantly elevated in the leaves of OE plants at 96 h post inoculation (2.9 times and 3.3 times that of wild type, respectively), suggesting a positive feedback regulation. Exogenous thiamine treatment (ET) also led to moderate induction of several thiamine-related genes, particularly in leaf tissue, though generally lower than in OE plants. Metabolite profiling showed that ThMP and thiamine levels in leaves were significantly increased in ET plants, while ThDP content was significantly elevated in OE plants compared to WT (Figure 6c). These changes were most prominent in leaf tissue, aligning with the observed gene expression trends. Collectively, these results indicate that both exogenous thiamine supplementation and *PvTPK* overexpression can enhance resistance to Fusarium wilt in susceptible plants, potentially through promoting thiamine accumulation and activating biosynthetic gene expression.

## 4. Discussion

THIC, TH1, THI1, and TPK are key enzymes involved in thiamine biosynthesis and play crucial regulatory roles in the synthesis of thiamine in plants [43]. In this study, bioinformatics approaches were employed to identify genes associated with thiamine biosynthesis at the whole-genome level in common bean. Except for the *PvTHI1* family, which contains two members (*PvTHI1-1* and *PvTHI1-2*), only a single gene was identified for each of *THIC*, *TH1*, and *TPK*. Phylogenetic and synteny analyses with other plant species (Figure 1 and Figure 2) revealed that neither tandem nor segmental duplications were detected for these gene families. This may account for the limited number of thiamine biosynthesis-related genes; however, the underlying molecular and evolutionary mechanisms responsible for this phenomenon warrant further investigation. The lack of gene duplication in the thiamine biosynthesis pathway suggests that these genes are functionally indispensable and highly conserved in the *P. vulgaris* genome. A similar pattern is observed in the model plant *Arabidopsis thaliana*, which contains only one gene each for *THIC*, *TH1*, and *THI1*, as well as two *TPK* family members, *TPK1* and *TPK2* [21,22]. In *Arabidopsis*, mutants of *THIC*, *TH1*, or *THI1* exhibit pale leaves and seedling lethality [17,18,44]. While single mutants of TPK do not show phenotypic differences from wild-type and can still synthesize thiamine, double mutants produce little or no TPP and also die at the seedling stage [20]. The low number and lack of duplication events for thiamine biosynthetic genes in *P. vulgaris* highlight their essential and evolutionarily conserved functions in plant development.

In recent years, increasing evidence has revealed that thiamine not only plays a critical role in plant growth and development but also contributes significantly to both biotic and abiotic stress responses [13,22,43]. To investigate whether thiamine is involved in common bean resistance to Fusarium wilt, we measured the expression levels of key thiamine biosynthetic genes and the endogenous thiamine content in both resistant and susceptible genotypes. The results showed that the expression levels of thiamine biosynthesis-related genes were significantly higher in the resistant genotype compared to the susceptible one, and the endogenous thiamine content was also markedly elevated in the resistant genotype (Figure 5). These findings suggest that thiamine may participate in the resistance response against Fusarium wilt in common bean. Currently, it is known that the mechanism by which thiamine mediates biotic stress responses is closely linked to the salicylic acid (SA) signaling pathway [24,27]. Pathogenesis-related (PR) proteins are induced by SA to trigger systemic acquired resistance (SAR) in plants. Our previous study demonstrated that the methyl esterase PvMES1 plays a role in common bean defense against Fusarium wilt by modulating the SA-mediated signaling pathway, including phenylpropanoid biosynthesis and sugar metabolism [32], indicating that resistance to Fusarium wilt in common bean is likely associated with SA signaling. Therefore, it is plausible that thiamine biosynthetic genes may be involved in SA-regulated defense responses against Fusarium wilt.

As a severe soil-borne disease affecting common bean, identifying effective strategies for resistance to Fusarium wilt is of great significance for disease control. Moreover, elucidating resistance mechanisms by regulating endogenous defense pathways through exogenous substances is important for combating this disease [45,46,47]. In this study, we found that the expression of thiamine biosynthesis-related genes and the endogenous thiamine content were significantly higher in resistant genotypes than in susceptible ones. Additionally, exogenous thiamine application promoted the biosynthesis of endogenous thiamine in common bean, thereby enhancing resistance to Fop. Previous studies have similarly shown that exogenous thiamine pretreatment leads to rapid and abundant accumulation of pathogenesis-related (PR) gene transcripts upon pathogen infection, thereby enhancing plant resistance. Furthermore, H_2_O_2_ has been found to be involved in the establishment of thiamine-induced plant defense systems [26]. Thiamine enhances antiviral immunity in maize by activating the MAPK pathway and promoting lignin biosynthesis, while Maize chlorotic mottle virus (MCMV) impairs this process by hijacking *ZmTHIC* to suppress thiamine synthesis [30]. Studies revealed that thiamine metabolism serves as a crucial intersection between biotic and abiotic stress responses in cotton, with *GhTHIC* and *GhTHI1* being strongly induced during the early stress stage. Further experiments confirmed that thiamine is essential for cotton growth and development, and its deficiency can be restored through exogenous supplementation. Moreover, exogenous thiamine enhances stress tolerance in cotton by strengthening calcium signaling and activating downstream stress-responsive genes [13]. These studies collectively demonstrate that exogenous thiamine can activate plant resistance by modulating the expression of endogenous thiamine biosynthetic genes. In this study, we selected the relatively less-studied but root-enriched gene *PvTPK* for overexpression in the susceptible common bean genotype using an *Agrobacterium rhizogenes*-mediated hairy root transformation system, which also conferred resistance to Fusarium wilt. *PvTPK* can serve as a core target gene for mining superior haplotypes of its promoter, coding region, and regulatory genes across diverse germplasm resources. Based on these sequence features, molecular markers can be developed for marker-assisted selection in breeding programs. Resistant genotypes exhibiting higher thiamine accumulation and upregulation of these genes under *Fop* infection can be directly incorporated into breeding pipelines. In addition, precise regulation of this pathway through genetic engineering approaches—such as overexpressing positive regulatory factors or editing negative regulatory elements—offers novel strategies for developing broad-spectrum and durable resistant varieties. Analysis of thiamine components revealed significantly increased ThDP accumulation—a key active coenzyme—in the roots of overexpression plants. Although thiamine biosynthesis mainly occurs in green tissues, it is required in all plant organs during development, and its specific role and localization in root-associated resistance remain unclear [48]. Hence, further mechanistic studies of this gene are expected to provide new insights into root-related thiamine-mediated defense.

## 5. Conclusions

In this study, thiamine biosynthesis-related genes were identified in the genome of common bean, and expression pattern analysis revealed that the thiamine biosynthetic pathway plays a critical role in resistance to Fusarium wilt. Furthermore, both exogenous application of thiamine to susceptible genotypes and overexpression of the key thiamine biosynthetic gene *PvTPK* significantly enhanced the expression of thiamine biosynthesis genes and promoted the accumulation of endogenous thiamine, thereby improving resistance to Fusarium wilt. While this study highlights the pivotal role of *PvTPK* in thiamine-mediated disease resistance, the complete thiamine metabolic pathway involves coordinated regulation by multiple genes such as *PvTHI1-1* and *PvTHIC*. Therefore, systematic validation of these genes’ functions in subsequent research will help comprehensively decipher the network mechanisms through which thiamine enhances plant immunity. These findings provide a foundation for future mechanistic studies of thiamine-mediated disease resistance in common bean and offer a potential strategy for its practical application in disease control.

## Figures and Tables

**Table 1:** The genomic and biochemical information for thiamine genes identified in common bean.

Gene Name	Locus ID	Chromosomal Location	DNA (bp)	Protein (aa)	MWb (Da)	pI	Subcellular Location
*PvTHIC*	Phvul.006G065300	Chr06:46364127-46368645	1950	650	72,414.16	6.1	chlo: 5, extr: 3, pero: 2
*PvTHI1-1*	Phvul.007G052900	Chr07:17589476-17596290	1065	355	37,832.6	5.93	chlo: 7.5, cyto: 6, chlo_mito
*PvTHI1-2*	Phvul.011G139100	Chr11:30895753-30901168	1044	348	36,750.34	5.85	chlo: 14
*PvTH1*	Phvul.006G216300	Chr06:4317738-4319440	1671	557	59,644.67	7.55	chlo: 11.5, chlo_mito: 7.5, mito: 2.5
*PvTPK*	Phvul.001G206100	Chr01:35496782-35499428	777	259	28,918.19	6.24	nucl: 5, cyto: 4, chlo: 3.5, chlo_mito: 3

## Data Availability

Data are contained within the article and Supplementary Materials.

## Supplementary Materials

The following supporting information can be downloaded at: https://www.mdpi.com/article/10.3390/biology14101366/s1, Table S1: Primers in this study; Table S2: List of the thiamine genes ID identified in this study; Table S3: Collinear gene pairs identified in common bean and other plants; Table S4: Ka/Ks value for the collinear gene pairs between common bean and other species; Table S5: *Cis*-regulatory elements in the promoter region of common bean in this study; Table S6: Classification criteria with element counts for *cis*-regulatory elements in promoter regions analyzed in this study; Table S7: Predictive transcription factor (TFs) analysis of thiamine biosynthesis genes.
